# Efficient Synthesis and HPLC-Based Characterization for Developing Vanadium-48-Labeled Vanadyl Acetylacetonate as a Novel Cancer Radiotracer for PET Imaging

**DOI:** 10.3390/molecules29040799

**Published:** 2024-02-09

**Authors:** Brittany A. Broder, Mohammed Bhuiyan, Richard Freifelder, David A. Rotsch, Satish K. Chitneni, Marvin W. Makinen, Chin-Tu Chen

**Affiliations:** 1 Department of Radiology, The University of Chicago, 5801 South Ellis Avenue, Chicago, IL 60637, USA; mohammedb@uchicago.edu (M.B.); makinen@uchicago.edu (M.W.M.); 2 National Institute of Standards and Technology, 100 Bureau Drive, Gaithersburg, MD 20899, USA; 3 Argonne National Laboratory, 9700 S Cass Avenue, Lemont, IL 60439, USA; rotschda@ornl.gov; 4 Oak Ridge National Laboratory, 5200, 1 Bethel Valley Road, Oak Ridge, TN 37830, USA

**Keywords:** vanadium, vanadium-48, vanadyl acetylacetonate, high performance liquid chromatography (HPLC), positron emission tomography (PET), PET imaging, radioactivity

## Abstract

*Bis*(acetylacetonato)oxidovanadium(IV) [(VO(acac)_2_], generally known as vanadyl acetylacetonate, has been shown to be preferentially sequestered in malignant tissue. Vanadium-48 (^48^V) generated with a compact medical cyclotron has been used to label VO(acac)_2_ as a potential radiotracer in positron emission tomography (PET) imaging for the detection of cancer, but requires lengthy synthesis. Current literature protocols for the characterization of VO(acac)_2_ require macroscale quantities of reactants and solvents to identify products by color and to enable crystallization that are not readily adaptable to the needs of radiotracer synthesis. We present an improved method to produce vanadium-48-labeled VO(acac)_2_, [^48^V]VO(acac)_2_, and characterize it using high-performance liquid chromatography (HPLC) with radiation detection in combination with UV detection. The approach is suitable for radiotracer-level quantities of material. These methods are readily applicable for production of [^48^V]VO(acac)_2_. Preliminary results of preclinical, small-animal PET studies are presented.

## 1. Introduction

The vanadyl (VO^2+^) cation is the most stable diatomic cation in chemistry, and VO(acac)_2_ is one of the most stable vanadyl chelates known, exhibiting an equilibrium association constant of the order of ~10^23^ [1,2]. It has been shown through application of electron nuclear double resonance (ENDOR) spectroscopy that VO(acac)_2_ enters cells as an intact metallochelate [3] while other, structurally similar vanadyl (VO^2+^) chelates of lesser stability dissociate in the blood stream, releasing VO^2+^ and the organic ligand [4]. Both the vanadyl cation and VO(acac)_2_ have long been associated with insulin-mimetic activity [5,6]. However, the structural basis of their activity, defined as increased cellular glucose uptake in insulin sensitive tissues, differs greatly: The action of VO^2+^ enhances glucose uptake into cells independently of the insulin receptor (IR) via activation of phosphatidyl 3’-kinase, that in turn enhances synthesis of glycogen from increased glucose uptake [7]. In contrast, VO(acac)_2_ promotes phosphorylation of tyrosine residues on the insulin receptor and the IR substrate-1 (IRS1) in a dose-dependent manner that is synergistic with added insulin [8]. The action of VO(acac)_2_ here is indirect, inhibiting protein tyrosine phospha-tase-1B (PTP1B), the enzyme that regulates the phosphotyrosine level of the IR [9]. VO(acac)_2_ is an uncompetitive inhibitor of PTP1B in the presence of phosphorylated analogs of its phosphotyrosine protein substrates [10].

In addition to the established role of PTP1B in glucose metabolism, PTP1B also regulates the phosphotyrosine level of other proteins and receptors that are, in general, regulators of cell migration, cell adhesion, and cellular proliferation. These include the human epidermal growth factor receptor-2 (HER2), the proto-oncogene tyrosine-protein kinase Src, the scaffolding adaptor protein ERBIN, the focal adhesion kinase FAK, and the cub domain containing protein-1 (CDCP1), among others [11,12]. PTP1B, thus, has a central role in cellular metabolism, proliferation, tumorigenesis, and anoikis. Gradual, increased accumulation of VO(acac)_2_ into neoplastic xenograft tissue, leading to a plateau in VO(acac)_2_ content, occurs over a time period of 3 h to 4 h following intravenous or intraperitoneal injection [3,13]. In contrast, the level of VO(acac)_2_ in neighboring, normal tissue increases immediately after injection, but washout of the metallochelate from normal tissue appears complete within 30 min following injection. The mechanism leading to sustained, high levels of VO(acac)_2_ in neoplasms in contrast to the more rapid release of the metallochelate from neighboring normal tissue is not defined. However, it is likely related to the cellular content of PTP1B as an inhibitor, for not only is VO(acac)_2_ preferentially sequestered in neoplastic tissue [3] but it also augments the uptake of 2-(Fluorine-18)-2-deoxy-glucose into xenograft tumors through a non-insulin dependent pathway [13].

Because we anticipate promising utility of VO(acac)_2_ as an inhibitor of PTP1B not only for cancer detection but also for monitoring neoplastic, tumorigenic processes lasting several days or weeks, we are investigating its potential as a radiotracer for PET imaging by labeling it with vanadium-48 (^48^V, t_1/2_ = 15.97 d). Because of the long half-life of ^48^V, cellular processes could be investigated for which more commonly used radioisotopes, e.g., ^18^F, t_1/2_ = 109.7 min; ^11^C, t_1/2_ = 20.4 min; ^15^O, t_1/2_ = 2.05 min, are unsuitable. In contrast to ^48^V being generated with dedicated solid targetry [14,15], in previous work, we developed a protocol to generate the radioisotope with use of a makeshift, solid target using the beamstop of a compact medical cyclotron [16]. Description of the synthesis of VO(acac)_2_ in the chemical literature is on the macroscale; characterization methods typically include visual inspection (by color) and crystallization and are unsuitable for radiotracer synthesis and characterization. We describe here the synthesis, purification, and characterization of the ^48^V-labled metallochelate. Activity levels are suitable for radiotracer studies generally employed in preclinical, small animal investigations. Preliminary results of these studies are presented. 

## 2. Results

### 2.1. [^48^V]VO(acac)_2_ Yield

In this study, the starting activity was 275 MBq (7.44 mCi). The overall decay-corrected yield of the 4-day synthesis was 12%; of the 232 MBq (6.26 mCi) potentially available at the end of synthesis, 28 MBq (0.77 mCi) of the radiotracer were produced. 

In the foil dissolution step, 90 MBq (2.42 mCi) could not be dissolved or leached from the foil, resulting in a step-wise yield of 65%. Further dissolution was not pursued due to time constraints. The AG 50w-X8 column separation yielded 162 MBq (4.38 mCi), accounting for 93% of the activity transferred (Certain commercial equipment, instruments, or materials are identified in this paper to foster understanding. Such identification does not imply recommendation by the authors or their affiliated institutions, nor does it imply that the materials or equipment identified are necessarily the best available for the purpose). To speed up sample evaporation, only two aliquots were chosen, totaling 154 MBq (4.16 mCi) of activity. After subsequent evaporation and refluxing steps (detailed in Section 2.1), a C-18 solid-phase-extraction cartridge (SPE) was used. A reaction sample of 143 MBq (3.87 mCi) was applied to the column, of which 108 MBq (2.92 mCi) passed through unbound, while 33 MBq (0.89 mCi) remained trapped in the cartridge. Elution yielded 27 MBq (0.77 mCi) in 400 μL of ethanol (EtOH); 2.3 MBq (0.061 mCi) was left in the round bottom flask, resulting in a step-wise yield of 19.9%. The entire synthesis required 4 d to complete. 

### 2.2. Isolating HPLC VO(acac)_2_ Signal from Acetylacetone

**Figure 1:**
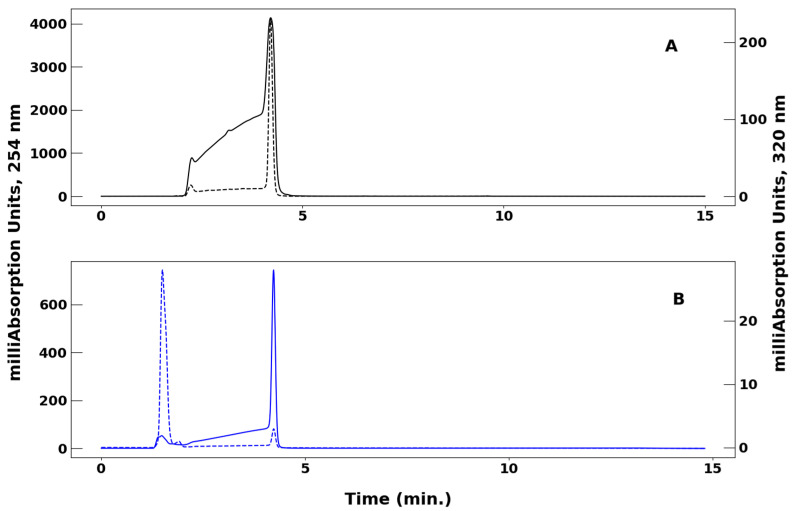
(**Panel A**): Effect of wavelength on retention time and chromatogram profile of acetylacetone monitored at 254 nm (solid) and 320 nm (dashed). (**Panel B**): Effect of wavelength on retention time and chromatogram profile of VO(acac)_2_ monitored at 254 nm (solid) and 320 nm (dashed). For both chromatograms, the solvent system was 40% to 60% MeCN/H_2_O containing 0.1% TFA and a flow rate of 1.5 mL/min.

Figure 1 shows chromatograms comparing acetylacetone and VO(acac)_2_ at 254 nm and 320 nm wavelengths. A gradient mobile phase of 40% to 60% acetonitrile (MeCN) in water with 0.1% Trifluoroacetic acid (TFA) was used. While acetylacetone and VO(acac)_2_ exhibit overlapping absorption in the far-UV at 254 nm (solid lines), the absorption properties for the two compounds are distinct at 320 nm (dashed lines). 

**Figure 2:**
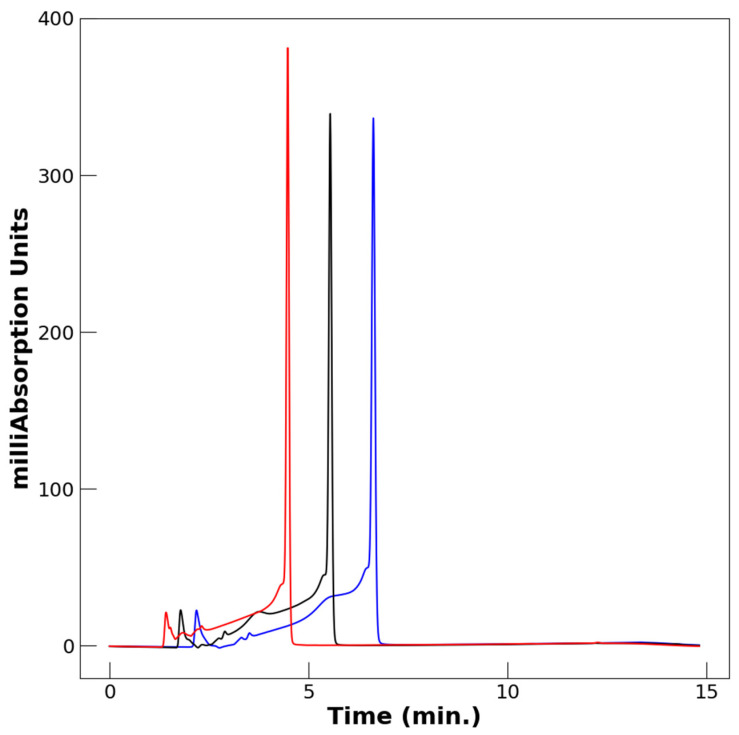
Effect of flow rate on retention time of VO(acac)_2_ monitored at 254 nm. Blue, 1.0 mL/min; black, 1.2 mL/min; red, 1.5 mL/min. The solvent system was 40% to 60% MeCN/H_2_O containing 0.1% TFA.

Figure 2 compares the effects of different flow rates. Of the three flow rates assessed for this mobile phase, 1.2 mL/min was chosen for further application. A smaller tail and a longer retention time are preferable; the chosen flow rate provided a middle ground of these conditions. 

### 2.3. Effect of Trifluoroacetic Acid

**Figure 3:**
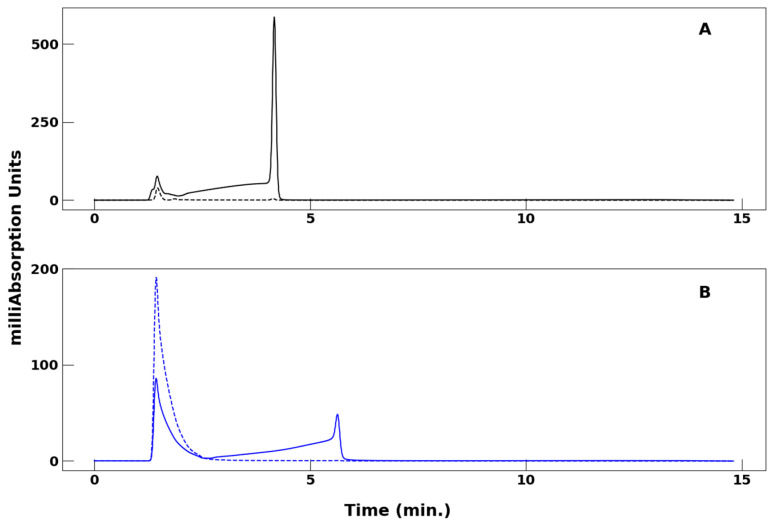
Comparison of HPLC of VO(acac)_2_ in a mobile phase of 40% to 60% MeCN/water in the presence and absence of TFA. (**Panel A**): Mobile phase containing 0.1% TFA monitored at 254 nm (solid line) and 320 nm (broken line). (**Panel B**): Same conditions as in (**Panel A**) but in the absence of TFA. For both panels, the flow rate was 1.2 mL/min.

Figure 3 compares chromatograms of VO(acac)_2_ in 40% to 60% MeCN/H_2_O in the presence and absence of TFA. In Figure 3A, the 320 nm trace exhibits a prominent peak at 1.47 min and almost no peak at 4.19 min, whereby the 1.47/4.19 ratio of the amplitudes is approximately 9:1. Correspondingly, the 254 nm trace illustrates a small feature at 1.47 min and a prominent peak at 4.19 min, whereby the 1.47/4.19 ratio of the amplitudes is interestingly 1:9. 

In Figure 3B, the 320 nm chromatogram shows a prominent feature with highest amplitude at 0.81 min followed by a tail. Similarly, the 254 nm trace shows a peak coinciding with that in the 320 nm trace at 0.81 min. It can be concluded that this feature belongs to the metallochelate. In the 254 nm trace, however, this peak is followed by a broad signal of lower amplitude starting at 2.79 min. Because there is no indication of the metallochelate at 2.79 min in the 320 nm trace, it does not contribute to this feature. 

### 2.4. Effect of Oxidation of the Metallochelate

**Figure 4:**
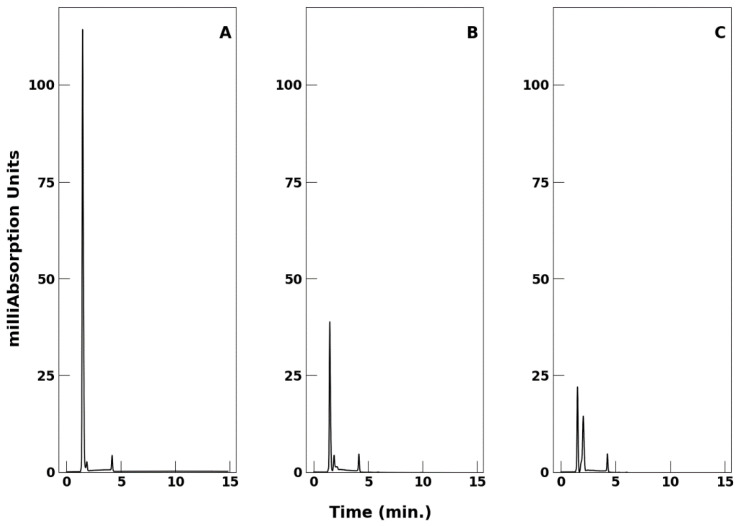
Comparison of HPLC of a sample of VO(acac)_2_ prepared immediately before use on day 1 and after standing at room temperature for 4 and 7 days. Chromatography was monitored at 320 nm, the flow rate was 1.5 mL/min, and the mobile phase was 40% to 60% MeCN/water. (**A**) Day 1; (**B**) day 4; (**C**) day 7.

Following the observations of Grybos et al. [17], who investigated the kinetics of oxidation of VO(acac)_2_ by molecular oxygen dissolved in methanol, we have carried out HPLC analysis of changes in the oxidation state of VO^2+^ moiety as a function of time. While the expectation is to use the radioactive product as quickly as possible upon synthesis of [^48^V]VO(acac)_2_, it is necessary to evaluate how changes in oxidation state over time may affect chromatographic behavior. The results are illustrated in Figure 4. 

In Figure 4A–C, a distinct peak appears in the HPLC chromatogram at 1.48 min. As a function of time, the amplitude of this peak decreases significantly by ~65% on day 4. Further decrease is observed a week after sample preparation. Additionally, other peaks emerge; we note that an additional species essentially immediately adjacent to the prominent 1.48 min component increases in amplitude with time, while the component at 4.03 min remains constant in amplitude. 

### 2.5. HPLC Comparison of Synthetic [^48^V]VO(acac)_2_ with Authentic VO(acac)_2_

**Figure 5:**
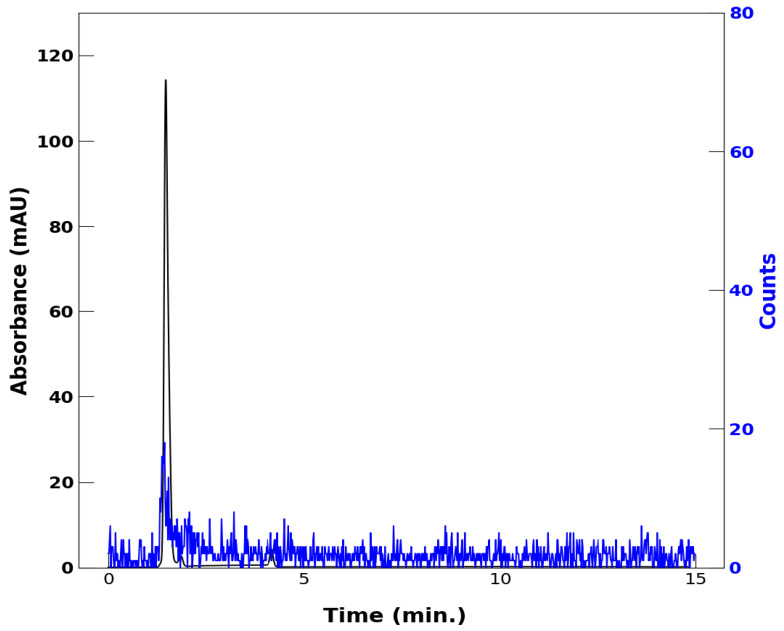
Comparison of HPLC of [^48^V]VO(acac)_2_ (blue trace) with that of authentic, commercially available VO(acac)_2_ (black trace). The retention time of the radiometallochelate is 1.49 min, while that of authentic VO(acac)_2_ is 1.56 min. The absorption of authentic VO(acac)_2_ was monitored at 320 nm. The vanadyl complexes were dissolved in 100% EtOH, and a 40% to 60% MeCN mobile phase was used with added 0.1% TFA.

Figure 5 compares the HPLC chromatogram to characterize [^48^V]VO(acac)_2_ synthesized as described in the Methods and Materials with that of an authentic sample of commercially available VO(acac)_2_ in 40% to 60% MeCN, as described in Figure 1, Figure 2, Figure 3 and Figure 4. The prominent peak of radioactivity due to [^48^V]VO(acac)_2_ showing a retention time of 1.49 min is essentially identical to that of 1.56 min of commercially available VO(acac)_2_ and confirms that the synthetic radiometallochelate is identical to the sample of authentic VO(acac)_2_. 

The maximum amplitude of the 320 nm peak in Figure 5 corresponds to 0.115 OD units. Extracting an extinction coefficient at 320 nm for VO(acac)_2_ from Figure 2 of Hon and coworkers [10] and applying it to the OD at 32 0nm of 0.115 absorbance units indicates that the 1 mL spectrophotometer cell of the Agilent 1260 Infinity II HPLC system contained 0.0051 mg of VO(acac)_2_. On the basis of a 5 μL injection volume of 2.0 mg of VO(acac)_2_ per mL EtOH, this represents 50% recovery of the commercially available metallochelate applied to the HPLC system. 

### 2.6. Results of Imaging Studies

**Figure 6:**
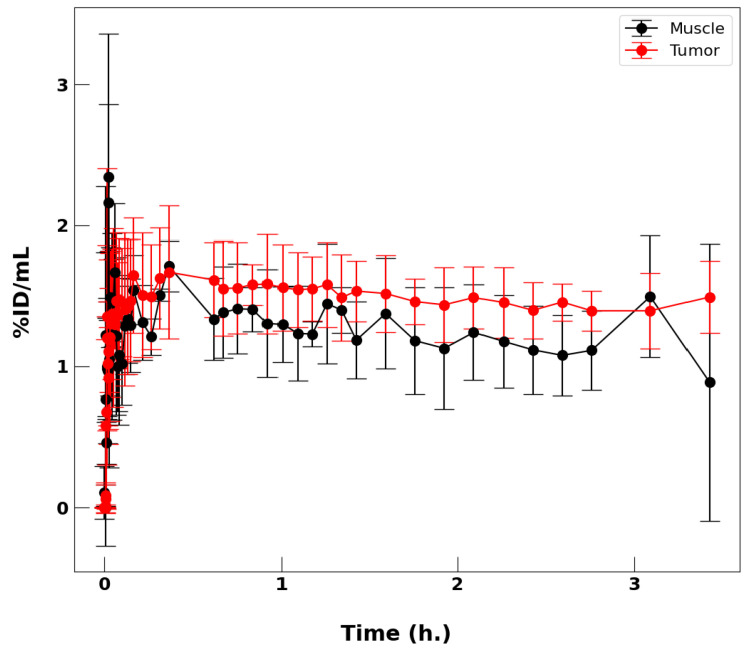
Comparison of radioactivity over time due to uptake of [^48^]VO(acac)_2_ into skeletal muscle and tumor tissue averaged over three mice.

In the three tumor-bearing HSD athymic mice with HCA-7 colonic adenocarcinoma cell lines that were imaged, tumor volumes averaged 101(52) mm^3^ and the injected dose was 2.81(0.24) MBq (75.90(6.49) μCi); uncertainties are the standard deviation of the mean. Figure 6 compares the time-activity curve for tumor tissue and skeletal muscle tissue from the contralateral leg. After approximately 30 min, the tumor tissue consistently had higher uptake than muscle tissue. The ratio between the two uptake values averages 1.23(0.85) over this time period (0.5 h to 4 h).

**Figure 7:**
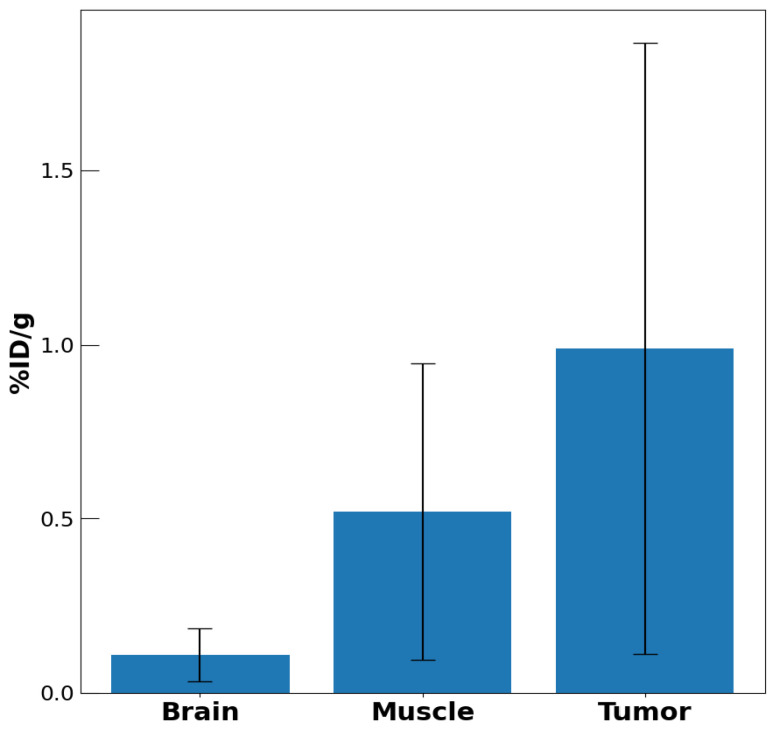
Biodistribution of radioactivity due to uptake of [^48^]VO(acac)_2_ into brain, skeletal muscle, and neoplastic tissue. The tissue was excised from each of the three mice after imaging for 4 h.

Figure 7 shows select biodistribution uptake values of [^48^V]VO(acac)_2_ in brain, skeletal muscle, and tumor tissue measured by ex-vivo gamma counting after imaging, again showing higher uptake in the tumor tissue compared to skeletal muscle tissue. The biodistribution data showed an average tumor to muscle ratio of 1.76(0.78), consistent with the last time point of Figure 6, exhibiting a ratio of 1.68(0.23). 

## 3. Discussion

### 3.1. [^48^V]VO(acac)_2_ Yield

Of the 232 MBq (6.26 mCi) potentially available at the end of synthesis, 28 MBq (0.77 mCi) was produced as the radiotracer. This yield of 12% is comparable to that of our previously reported method (13%) [16]. However, the method presented herein has the distinct advantage of being completed in a much shorter time frame (4 days compared to 13 days, respectively). This improved timescale allows for higher radiation yields and provides flexibility in down-stream radiotracer utility, making it preferable for future work. 

### 3.2. Isolating HPLC VO(acac)_2_ Signal from Acetylacetone

HPLC analysis of VO(acac)_2_ and acetylacetone using a gradient mobile phase of 40% to 60% MeCN in water with 0.1% TFA showed overlapping absorption in the far-UV at 254 nm (Figure 1, solid lines), a wavelength commonly used in HPLC characterization of organic compounds. Further studies revealed that the absorption properties for the two compounds are distinct at 320 nm. This indicates that the shared peak visible at 254 nm can be attributed to de-complexed acetylacetone. 

For this method, a flow rate of 1.2 mL/min was determined to produce the best elution profile. In comparison, Figure 2 demonstrates that a flow rate of 1.0 mL/min had a slightly longer retention time but a slightly larger tail, while a flow rate of 1.5 mL/min had a slightly shorter retention time and a slightly smaller tail. While the ideal profile would have the smallest tail, the flow rate that produced that condition, 1.5 mL/min, also had a shorter retention time and was therefore not adopted. 

### 3.3. Effect of Trifluoroacetic Acid

The comparison of HPLC chromatograms with and without TFA (Figure 3) illustrates that the presence of TFA enhances the peak shape of VO(acac)_2_. TFA was shown to impact the HPLC profile by sharpening peaks, leading to more precise retention times. In the chromatograms in which TFA is absent (Figure 3B), a peak is observed for both the 254 nm and 320 nm wavelengths at 0.81 min, which can be attributed to the VO(acac)_2_ metallochelate. However, the 254 nm trace, on the other hand, shows an additional broad signal of lower amplitude starting at 2.79 min. Because there is no indication of the metallochelate at 2.79 min in the 320 nm trace, it does not contribute to this feature. Further study is needed to understand the difference in chromatogram structure at these two wavelengths, which is currently being carried out in our laboratories. 

Although there are no direct structural data to explain the basis of the interaction of TFA with VO(acac)_2_, it is possible that the effect is not through acidification of the mobile phase despite the low acid dissociation constant (pKa ~ 0.25) of TFA in water [18]. The vanadyl chelate is known to dissociate under acidic conditions, yielding the free VO^2+^ cation [19]. 

A suggestion of how TFA improved resolution can be made on the basis of the structure of VO(acac)_2_ in aqueous methanol [20] and the greatly depressed pKa (~12.65) of TFA in MeCN [21]. Determination of the structure of VO(acac)_2_ in aqueous methanol by ENDOR spectroscopy revealed a methanol molecule hydrogen bonded to the oxygen atom of the VO^2+^ moiety [20]. Under the conditions that the pKa of TFA is depressed in aqueous MeCN mixtures, it is possible that the TFA molecule is hydrogen bonded to the vanadyl oxygen through its undissociated carboxylic acid group. Complex formation through hydrogen bonding of TFA to the vanadyl oxygen atom would change the hydrodynamic properties of the metallochelate, resulting in its separation from acetylacetone. Future work could use a milder acid in order to ensure compound integrity and maintain sharp absorption peaks. 

### 3.4. Effect of Oxidation of the Metallochelate 

HPLC analysis of a VO(acac)_2_ sample (Figure 4) demonstrated the effects of oxidation. Over the course of a week, the amplitude of the primary signal decreased. Because oxidation of the VO^2+^ moiety in VO(acac)_2_ results in decreased absorption in the 310 nm to 320 nm region with concomitant increase in absorption intensity in the 270 nm region [10], the decrease in amplitude of the 1.48 min feature is likely due entirely to oxidation of V^4+^ to the V^5+^ oxidation state.

Additionally, during this 1-week period, several other peaks appeared in the chromatogram. The additional species adjacent to the prominent 1.48 min component increased in amplitude with time, while the component at 4.03 min remained constant in amplitude. There are no structural data to characterize the oxidized species further except to note the change in oxidation state. Because oxidized species of the metallochelate exhibit altered inhibitory properties with respect to the kinetics of protein tyrosine phosphatase-1B catalyzed reactions compared to those in the presence of VO(acac)_2_ [10], the oxidized and reduced species must differ from each other in structure.

### 3.5. HPLC Comparison of Synthetic [^48^V]VO(acac)_2_ with Authentic VO(acac)_2_

In the HPLC chromatograms of both the commercially available compound and the synthesized radiotracer (Figure 5), the prominent peaks were shown to correspond quite well, indicating that the synthetic radiometallochelate was identical to the sample of authentic VO(acac)_2_. The strong overlap between the black and the blue traces in Figure 5 indicates that the radiometallochelate synthesized in the presence of EtOH has maintained the VO^2+^ moiety in its +4 oxidation state.

### 3.6. Results of Imaging Studies

Animal imaging and biodistribution studies showed a higher uptake in tumor tissue compared to muscle. However, the standard deviation was high for tumor to muscle ratios of both studies: 15% for the last point in the time-activity curve in Figure 6 and 44% for the biodistribution study in Figure 7. This somewhat high value reflects a large variation in uptake of radioactivity among the three mice imaged. Further animal imaging studies are needed to determine more accurately the time dependent uptake of the radiometallochelate into different tissues and to assess the applicability of this radiotracer for small animal imaging studies.

## 4. Materials and Methods 

### 4.1. [^48^V]VO(acac)_2_ Synthesis

Chemicals and reagents of the highest purity available were purchased from Sigma-Aldrich (St. Louis, MO 63103, USA). Except for acetylacetone, no further purification of commercially available reagents was carried out. Acetylacetone was treated first with a mass concentration of 4% NaHCO_3_ to remove acetic acid, followed by brine, drying over Na_2_SO_4_, and finally filtration with a fritted glass funnel under partial vacuum. For HPLC, an Agilent 1260 Infinity II system equipped with a 250 mm × 4.6 mm Phenomenex Luna 5 μm C18(2) 100 Å column was used. Milli-Q deionized water at 18 MΩ was used throughout.

**Scheme 1:**
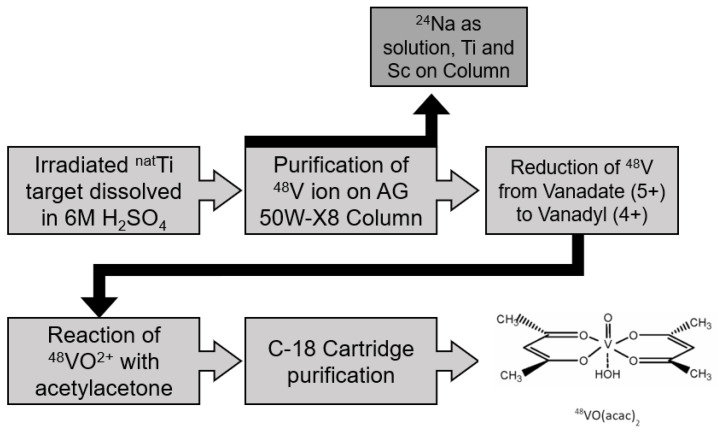
Diagram of synthesis of [^48^V]VO(acac)*_2_*.

The efficient synthesis of [^48^V]VO(acac)_2_ was based on the procedures described by Rowe et al. [22] and Zeisler et al. [15]. A summary is shown below in Scheme 1. The ^48^V was produced by irradiating natural titanium foils with 18 MeV protons in the beamstop of an IBA Cyclone^®^ 18/9 cyclotron (IBA, Louvain-La-Neuve, Belgium) for approximately 40 h over 5 d [16]. The irradiated foils were weighed and placed in a perfluoro-alkoxy-coated beaker over an oil bath at 140 °C and dissolved by addition of 2 mL 6 mol/L H_2_SO_4_. A column was prepared with 3.0 g of AG 50W-X8 cation exchange resin (mesh size 100 μm to 200 μm) and fitted with a frit. The target solution was diluted to 30 mL by addition of 28 mL H_2_O. It was then loaded onto the preconditioned column. The column was eluted with a solution of 1% H_2_O_2_ in 0.01 mol/L HNO_3_ and collected in 1 mL aliquots. Aliquots containing radioactivity were combined and the sample was evaporated by adding 100 μL concentrated H_2_SO_4_ and heating overnight. 

After cooling to room temperature, 1 mL EtOH, 0.5 mL H_2_O, and 0.05 mL 0.2 mol/L H_2_SO_4_ were added with refluxing overnight to reduce vanadium from oxidation state +5 to oxidation state +4. The next day, 0.5 mL acetylacetone was added with shaking. The solution was refluxed for 1 h further and then allowed to cool to room temperature after which the solution was neutralized with 0.8 g Na_2_CO_3_ dissolved in 5 mL H_2_O. The mixture was then applied to a C-18 SPE cartridge preconditioned with 5 mL EtOH, followed by addition of 10 mL water. The trapped [^48^V]VO(acac)_2_ was eluted in 400 μL EtOH. 

### 4.2. Characterization of [^48^V]VO(acac)_2_

While HPLC chromatography can be used to separate the metallochelate from excess acetylacetone, in this investigation, it was used to characterize the product. After synthesis, the product was characterized by both gamma ray spectroscopy with use of a NaI(Tl) detector and reverse-phase HPLC with 40% to 60% gradient of MeCN in water mobile phase over 10 min, 1 min at 60% MeCN, decrease to 40% MeCN in 1 min, and remaining at 40% MeCN for 3 min. Other gradients and mobile phases were considered in selecting this protocol; a complete summary has been made by Broder [23]. TFA (volume concentration 0.1%) was added to the water phase to improve peak shape. TFA at this concentration has been shown to exhibit no absorption at 254 nm in MeCN/water [24], the wavelength at which acetylacetone was monitored spectrophotometrically. Commercially available VO(acac)_2_ was dissolved in 100% EtOH at a concentration of 2 mg/mL. Acetylacetone was dissolved in EtOH at a concentration of 5.4 mg/mL. An injection volume of 5 μL corresponding to 0.01 mg VO(acac)_2_ or 0.027 g acetylacetone, respectively, was used. Solutions of VO(acac)_2_ were freshly prepared unless otherwise noted. In some cases, freshly prepared solutions were compared to older samples (age of preparation < 1 week) to determine possible effects of oxidation of the metallochelate on its chromatographic behavior. 

A flow rate ranging from 1.0 to 1.5 mL/min was used resulting in approximately 15 min per trial chromatographic separation. The eluent was monitored optically at 254 nm for acetylacetone and at 320 nm for VO(acac)_2_ unless otherwise specified [10].

### 4.3. Imaging Studies with [^48^V]VO(acac)_2_

HSD athymic, female, nude mice were acquired from Harlan-Envigo (Indianapolis, IN 46250, USA) and three mice were inoculated subcutaneously with 1 × 10^6^ to 5 × 10^6^ HCA-7 cells in the right hind limb. Tumors were allowed to grow for up to 6 weeks until a tumor volume > 100 mm^3^ was reached. 

[^48^V]VO(acac)_2_ was prepared by evaporating the solution to dryness and reconstituting it in normal saline. Prior to imaging studies, mice were anesthetized and injected via the tail vein with approximately 2.8 MBq (75 µCi) of [^48^V]VO(acac)_2_ in normal saline. Animals were imaged for 4 h using a β-Cube PET system (Molecubes, Lexington, MA 02421, USA). Computed tomography (CT) scans were obtained after PET imaging. After 4 h, the animals were sacrificed and biodistribution studies were performed. All tissues were assayed with the use of a gamma counter. Time-activity curves were obtained using Vivo-Quant (Invicro, Needham Heights, MA 02494, USA). Regions of interest (ROIs) were manually selected per animal based on the CT scan and confirmed with the PET image to measure the activity in the organ as a function of time.

All animals were handled and studies were conducted in accordance with protocols approved by The University of Chicago Institutional Animal Care and Use Committee.

## 5. Conclusions

In this investigation, we have described the synthesis of [^48^V]VO(acac)_2_, a novel radiotracer, and characterized it according to its HPLC chromatographic behavior. This more efficient synthesis method, requiring only four days to complete, provided a yield of 27 MBq. Although the yield was comparable to that of 32 MBq ± 3 MBq in our previous study that required approximately three weeks for preparation of the radiometallochelate [16], the present method requiring only four days represented a substantial time improvement. Illustrative preclinical imaging studies demonstrated higher time dependent uptake of [^48^V]VO(acac)_2_ in xenograft tumor tissue compared to that in skeletal muscle tissue. Further investigation is needed to accurately determine the maximum uptake of the radiotracer in xenograft tumors over time and in all organs for a complete biodistribution study. 

## Data Availability

The data presented in this study are available on request from the corresponding authors. The data are not publicly accessible because they are not available in a format that is sufficiently accessible or reusable by other researchers.

## References

[1] Martell A.E., Smith R.M. (1977). Critical Stability Constants.

[2] Chasteen N.D., Berliner L.J., Reuben J. (1981). Vanadyl(IV) EPR Spin Probes: Inorganic and Biochemical Aspects. Biological Magnetic Resonance.

[3] Mustafi D., Peng B., Foxley S., Makinen M.W., Karczmar G.S., Zamora M., Ejnik J., Martin H. (2009). New vanadium-based magnetic resonance imaging probes: Clinical potential for early detection of cancer. J. Biol. Inorg. Chem..

[4] Setyawati I.A., Thompson K.H., Yuen V.G., Sun Y., Battell M., Lyster D.M., Vo C., Ruth T.J., Zeisler S., McNeill J.H. (1998). Kinetic analysis and comparison of up-take, distribution, and excretion of V-48-labeled compounds in rats. J. Appl. Physiol..

[5] Crans D.C., Smee J.J., Gaidamauskas E., Yang L. (2004). The Chemistry and Biochemistry of Vanadium and the Biological Activities Exerted by Vanadium Compounds. Chem. Rev..

[6] Thompson K.H., McNeill J.H., Orvig C. (1999). Vanadium Compounds as Insulin Mimics. Chem. Rev..

[7] Pandey S.K., Anand-Srivastava M.B., Srivastava A.K. (1998). Vanadyl Sulfate-Stimulated Glycogen Synthesis Is Associated with Activation of Phosphatidylinositol 3-Kinase and Is Independent of Insulin Receptor Tyrosine Phosphorylation. Biochemistry.

[8] Ou H.S., Yan L.M., Mustafi D., Makinen M.W., Brady M.J. (2005). The vanadyl (VO^2+^) chelate bis(acetylacetonato)oxo-vanadium(IV) potentiates tyrosine phosphorylation of the insulin receptor. J. Inorg. Biol. Chem..

[9] Elchebly M., Payette P., Michaliszyn E., Cromlish W., Collins S., Loy A.L., Nor-mandin D., Cheng A., Himms-Hagen J., Chan C.C. (1999). Increased insulin sensitivity and obesity resistance in mice lacking the protein tyrosine phosphatase-1B gene. Science.

[10] Hon J., Hwang M.S., Charnetzki M.A., Rashed I.J., Brady P.B., Quillin S., Makinen M.W. (2017). Kinetic characterization of the inhibition of protein tyrosine phosphatase-1B by Vanadyl (VO^2+^) chelates. J. Biol. Inorg. Chem..

[11] Mertins P., Eberl H.C., Renkawitz J., Tremblay M.L., Mann M., Ullrich A., Daub H. (2008). Investigation of Protein-Tyrosine Phosphatase 1B function by Quantitative Proteomics. Mol. Cell. Proteomics.

[12] Bonham C.A., Mondati V., Singh R.K., Pappin D.J., Tonks N.K. (2023). Coupling substrate-trapping with proximity-Labeling to identify protein tyrosine phosphatase PTP1B signaling networks. J. Biol. Chem..

[13] Makinen M.W., Bamba R., Ikejimba L., Wietholt C., Chen C.T., Conzen S.D. (2013). The vanadyl chelate bis(acetyl-acetonato)oxovanadium(IV) increases the fractional uptake of 2-(fluorine-18)-2-deoxy-D-glucose by cultured human breast carcinoma cells. Dalton Trans..

[14] Bonardi M.L., Rizzio E., Gallorini M., Groppi F., Mainardi H.S. (2005). Improved radiochemical separation of no-carrier-added vanadium-48 from proton irradiated titanium target. J. Radioanal. Nucl. Chem..

[15] Zeisler S.K., Ruth T.J. (1995). Preparation of [48V]-VO^2+^ For Biomedical Studies. J. Radioanal. Nucl. Chem. Lett..

[16] Broder B.A., Bhyuian M.P., Freifelder R., Zhang H.J., Kucharski A., Makinen M.W., Rotsch D.A., Chen C.-T. (2022). Preliminary Investigation of 48V-labeled VO(acac)_2_ for Cancer Imaging: An Initial Proof-of-Concept Study. Appl. Radiat. Isot..

[17] Grybos R., Samotus A., Popova N., Bogolitsyn K. (1997). Kinetics of oxidation of vanadyl acetylacetonate by oxygen in methanolic solution. Transit. Met. Chem..

[18] Dean J.A. (1973). Lange’s Handbook of Chemistry.

[19] Correia I., Chorna I., Cavaco I., Roy S., Kuznetsov M.L., Ribeiro N., Justino G., Marques F., Santos-Silva T., Santos M.F. (2017). Interaction of [VIVO(acac)_2_] with human serum transferrin and albumin. Chem.–Asian J..

[20] Mustafi D., Makinen M.W. (2005). Structure and Conformation of Bis(acetylacetonato)oxovanadium(IV) and Bis(mal-tolato)oxovandium(IV) in Solution Determined by Electron Nuclear Double Resonance Spectroscopy. Inorg. Chem..

[21] Oldacrea A.N., Young E.R. (2020). Investigation of Electrochemical Proton-Coupled Electron Transfer of Anthracene-based Azo dye. Electronic Supplementary Material (ESI) for RSC Advances.

[22] Rowe R.A., Jones M.M. (1957). Preparation from vanadium(v) oxide through prior reduction to oxovanadium(iv) ion. Inorg. Synth..

[23] Broder B.A. (2022). Development and Kinetic Analysis of Emerging Positron Emission Tomography Radiotracer Vandium-48-Labeled Vanadyl Acetylacetonate. Ph.D. Dissertation.

[24] Winkler G., Wolschann P., Briza P., Heinz F.X., Kunz C. (1985). Spectral Properties of Trifluoroacetic Acid-Acetonitrile Gradient Systems for Separation of Picomole Quantities of Peptides by Reversed-Phase High Performance Liquid Chromatography. J. Chromatog..

